# An R124C mutation in TGFBI caused lattice corneal dystrophy type I with a variable phenotype in three Chinese families

**Published:** 2008-06-30

**Authors:** Zhe Liu, Yi-qiang Wang, Qing-hua Gong, Li-xin Xie

**Affiliations:** 1 Department of Ophthalmology, Medical College of Zhejiang University, Hangzhou, China; 2 Eye Hospital, Wenzhou Medical College, Wenzhou, China; 3 State Key Laboratory Cultivation Base, Shandong Provincial Key Laboratory of Ophthalmology, Shandong Eye Institute, Qingdao, China

## Abstract

**Purpose:**

A genetic and clinical study of three unrelated Chinese pedigrees with a variable phenotype of lattice corneal dystrophy type I (LCD I).

**Methods:**

The eyes of the patients were examined by slit lamp microscopy, and other clinical records were also collected. Genomic DNA was extracted from peripheral leukocytes of the affected patients and their family members. Exons of the transforming growth factor β induced *TGFBI* gene were amplified by polymerase chain reaction and directly sequenced to verify the mutation. Fifty healthy volunteers were analyzed as normal controls.

**Results:**

Variable atypical clinical features of LCD I were found by slit lamp microscopy in these three Chinese pedigrees. A heterozygous single base-pair transition from C to T (C417T), leading to amino acid substitution (R124C) in the encoded TGFBI protein, was detected in all of the affected patients. No mutation was found in unaffected family members and 50 normal controls.

**Conclusions:**

Clinical features of Chinese patients with the same R124C mutation are quite variable even within the same family. Molecular genetic analysis of *TGFBI* can offer a rapid, accurate diagnosis of patients with atypical corneal dystrophies.

## Introduction

Lattice corneal dystrophy type I (LCD I; OMIM 122200) is an autosomal dominantly inherited corneal amyloidosis that is characterized by thin branching refractive lines in the anterior corneal stroma, leading to progressive opacification, painful bilateral recurrent corneal erosions, and severe visual defect. LCD I always manifests itself in the first few decades of life especially within the second decade. Mutations in the human transforming growth factor β induced (*TGFBI*) gene are the main causes of LCD I [1].

*TGBFI* is located on the long arm of chromosome V (5q31) and encodes for the adhesion molecule [2], which is an extracellular matrix adhesion protein inducible by transforming growth factor β (TGFBIp). *TGBFI* was first isolated by Skonier et al. [3]. It is a prominent protein in the cornea, skin, and matrix of many connective tissues. Based on the molecular genetic study of 5q31-linked autosomal dominant corneal dystrophy, the correspondence of the genotype-phenotype has been recognized that specific mutation causes the defined form of corneal dystrophy CD [4]. Molecular genetic studies of 5q31-linked corneal dystrophies have demonstrated a clear genotype-phenotype correlation as specific *TGFBI* gene mutations cause defined forms of CD. For example, R124C has been identified as the most frequent mutation associated to LCD I throughout the world [5-11].

Here, we report three unrelated Chinese families with the identical mutation of R124C in TGFBIp. Two families are Han Chinese, another is a descendant of Han and Inner Mongolia. Patients in these families show remarkable variable phenotypes of LCD I. The facts indicate that, with the exception of the typical form of LCD I with the R124C mutation described before, Chinese LCD I patients with R124C mutation also have variable atypical clinical features even within the same family. This is the first report of the phenotypic variability of the R124C mutation in Chinese LCD I pedigrees.

## Methods

Informed, written consents were obtained from all participants according to the Declaration of Helsinki. The study was approved by the internal board of the Shandong Eye Institute (Qingdao, China).

**Figure 1:**
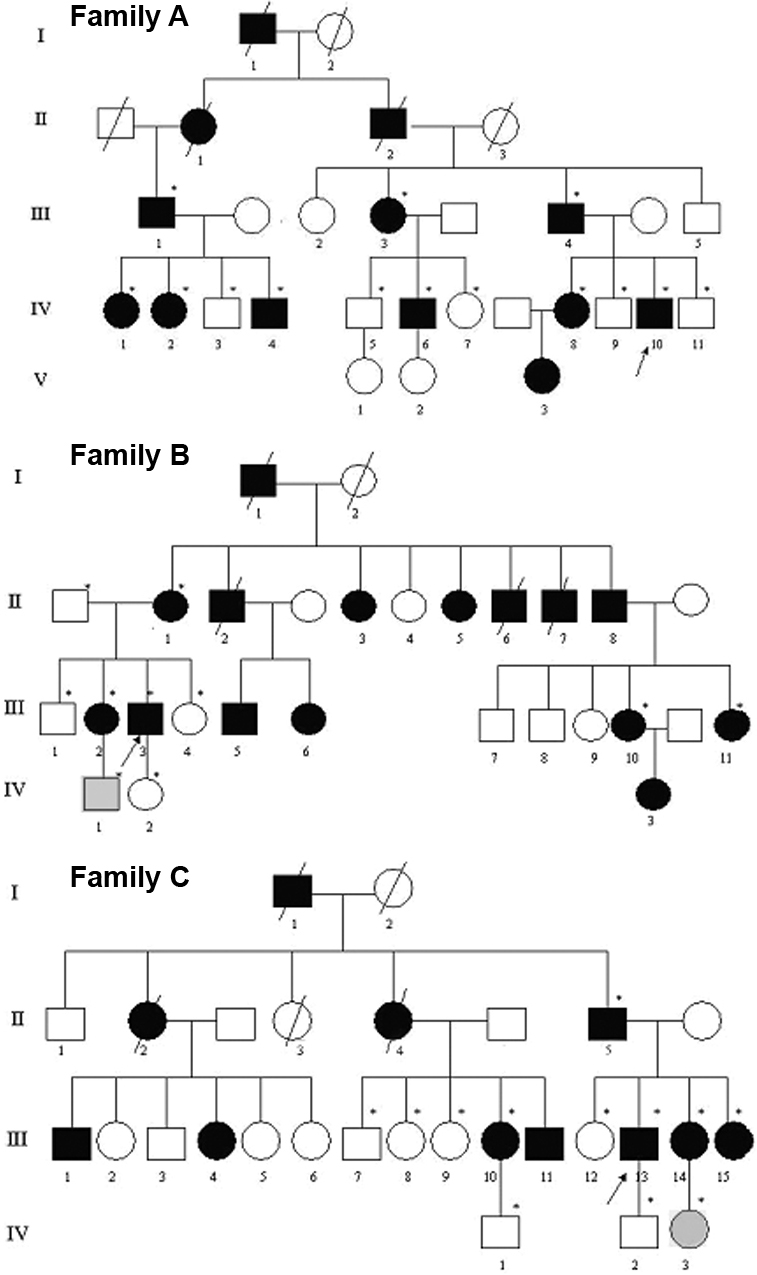
The family hereditary patterns of three Chinese pedigrees. The pedigrees show autosomal dominant transmission of the disease. The arrows indicate the probands, the asterisks indicate subjects who underwent clinical and molecular analyses, black symbols represent affected subjects, and gray symbols represent young subjects who carry the same mutation but still remain asymptomatic.

Three unrelated Chinese pedigrees of LCD were obtained for our study (Figure 1). Thirty-six family members (19 patients, 15 unaffected and 2 young asymptomatic relatives) from these three Chinese families participated in our study. Fifty age-matched healthy Chinese volunteers participated in the study as normal controls. Control subjects were recruited from medical students, hospital staff members, and residents. All of control subjects had a negative family history for LCD with negative history for ophthalmic disease and without any systemic diseases.

### Ophthalmologic examinations

Complete ophthalmologic examinations were performed on the probands and other patients of these three Chinese families. The patients underwent vision examination, slit lamp biomicroscopy, applanation tonometry, fundus examination, and anterior segment photography.

### Genetic analysis

Genomic DNA was isolated from the whole blood of participants by the standard phenol chloroform method. *TGFBI* was analyzed by direct genomic DNA sequencing of exons 4, 10, 12, and 14. The forward and reverse oligonucleotide primers were described previously [5]. Polymerase chain reaction (PCR) was performed in a volume of 50 μl in a DNA Thermal Cycler (Perkin Elmer, Norwalk, CT). PCR conditions were as follows: 5 min at 94 °C followed by 30 cycles of 94 °C for 1 min, 60 °C for 1 min, and 72 °C for 1 min with a final extension step at 72 °C for 10 min.

Each PCR fragment was purified (QIAquick, Qiagen, Hilden, Germany), and then both strands were subsequently analyzed by direct sequencing in an ABI 3700 automated DNA sequencer using the Big Dye Terminator Cycle sequencing reaction kit (Perkin-Elmer, Applied Biosystems Division, Foster City, CA). Sequence results were compared with the wild type *TGFBI* sequence (GenBank AY149344). The other family members and 50 Chinese controls were screened for the presence of R124C mutation by PCR amplification and then by direct sequencing of PCR products.

## Results

### Clinical evaluation

**Figure 2:**
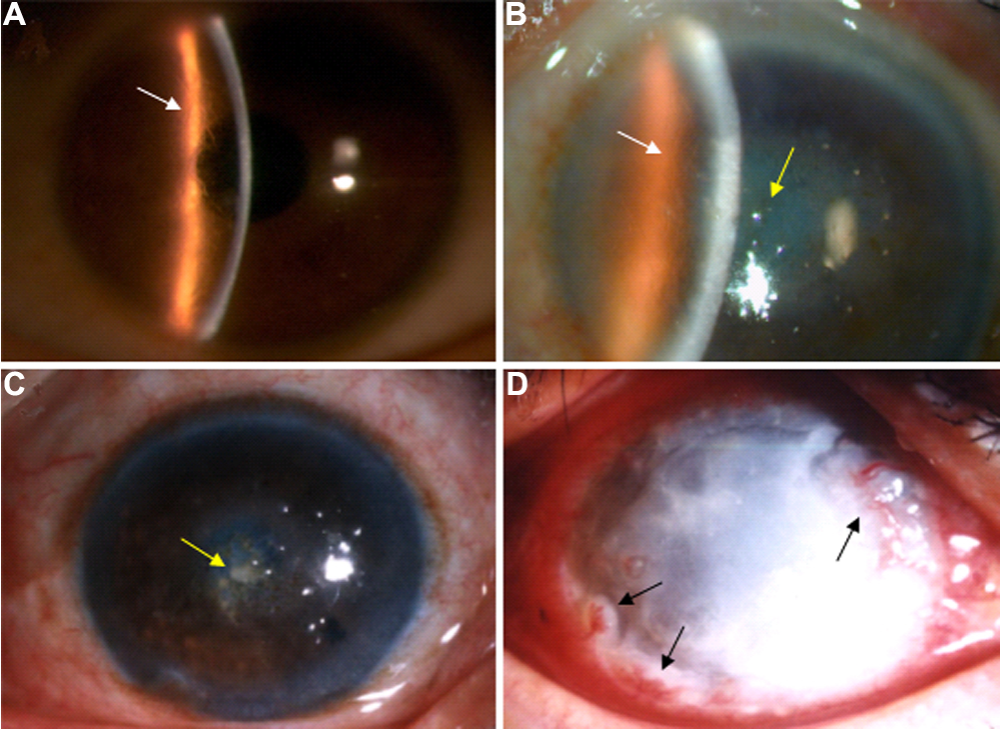
Slit lamp photographs of the affected family members with LCD I due to an R124C mutation in *TGFBI*. **A**: The eye of proband in family A (IV-10) shows the typical lattice lines of LCD I (white arrow). **B** and **C**: III-4 of family A (**B**) and II-5 of family C (**C**) show diffuse opacity occupying the central cornea with superimposed amorphous deposits (yellow arrow), which is quite different from typical LCD I. III-4 from family A also manifested thin branching refractive lines in the anterior corneal stroma (white arrow). **D**: The eye of the proband of family B (III-3) shows a diffuse opacity throughout the cornea, and no lattice lines could be found. There is also neovascular infiltration from the limbus of the cornea (black arrows).

The transmission patterns of these three Chinese pedigrees are consistent with autosomal dominant inheritance (Figure 1). General clinical data of patients from the three families were shown in Table 1, Table 2, and Table 3. The average age of onset was 20, 19, and 19.8 years old, respectively. Some patients such as IV-10, the proband of family A, showed typical clinical features of LCD I (Figure 2A). He was 20 years old when he had bilateral recurrent corneal erosions and progressive visual defect. Slit lamp examination of both eyes revealed thin branching refractive lines in the anterior corneal stroma. The small lattice-shaped opacity is the characteristic feature of LCD I, which can be observed by direct illumination and retroillumination. Such typical clinical features of some patients illustrated the diagnosis of LCD I in these three families.

**Table 1:** Clinical data for the affected individuals of family A with TGFBIp mutation R124C.

**Patient number/ Sex/Age (years)**	**Age of onset (years)**	**Time of duration (years)**	**Surgical treatment (patient’s age when performed)**	**Visual acuity (before surgery)**	**Visual acuity (after surgery)**
III-1/M/72	22	50	OU: ND	OU: HM	—
III-3/F/69	24	45	OU: PKP (59)	OU: FC/30cm	0.1
III-4/M/67	27	40	OD: LKP (57)	OD: 0.06	OD: 0.4
			OS: PKP (57)	OS: 0.1	OS: 0.4
IV-1/F/40	20	20	OU:PKP (37)	OD: 0.15	OD: 0.4
				OS: 0.3	OS: 0.5
IV-2/F/38	21	17	OU: ND	OD: 0.6	—
				OS: 0.1	—
IV-4/M/35	19	16	OD: PKP (34)	OD: 0.1	OD: 0.5
			OS: ND	OS: 0.2	—
IV-6/M/41	21	20	OD: PKP (31)	OD: 0.05	OD: 0.4
			OS: ND	OS: 0.4	—
IV-8/F/49	18	31	OU: ND	OU: 0.5	—
IV-10/M/41	35	20	OD: PKP (33)	OD: 0.04	OD: 0.4
			OS: *	—	—
V-3/F/20	19	1	OU: ND	OU: 0.8	—

The Snellen visual acuity scale was used in these studies. The asterisk indicates that the patient’s left eye is an artificial eye resulting severe trauma in childhood. HM: hand movement; FC: finger count; LKP: lamellar keratoplasty; PKP: penetrating keratoplasty; ND: not done keratoplasty.

**Table 2:** Clinical data for the affected individuals of family B with TGFBIp mutation R124C.

**Patient number/ Sex/Age (years)**	**Age of onset (years)**	**Time of duration (years)**	**Surgical treatment (Patient’s age when performed)**	**Visual acuity (before surgery)**	**Visual acuity (after surgery)**
II-1/F/70	20	50	OU: ND	OU: HM	—
II-3/F/66	18	48	OU: PKP (44)	OU: FC/40cm	OU: 0.1
III-2/F/47	22	25	OU: ND	OU: 0.4	—
III-3/M/43	13	30	OD:PKP (31)	OD: HM	OD: 0.4
			OS: ND	OS: 0.5	—
III-5/M/46	21	25	OD: PKP (34)	OD: 0.15	OD: 0.4
			OS: ND	OS: 0.2	—
III-10/F/43	20	23	OU: ND	OD: 0.5	—
				OS: 0.1	—
III-11/F/40	20	20	OU: ND	OU: 0.3	—
IV-3/F/20	19	1	OU: ND	OU: 1.0	—

**Table 3:** Clinical data for the affected individuals of family C with TGFBIp mutation R124C.

**Patient number/Sex/ Age (years)**	**Age of onset (years)**	**Time of duration (years)**	**Surgical treatment (Patient’s age when performed)**	**Visual acuity (before surgery)**	**Visual acuity (after surgery)**
II-5/M/67	17	60	OU: PKP (62)	OU: HM	OU: 0.2
III-10/F/35	19	16	OU: ND	OU: 0.5	—
III-11/M/32	22	10	OU: ND	OU: 0.6	—
III-13/M/40	25	15	OU: ND	OU: 0.7	—
III-14/F/37	12	25	OU: ND	OU: 0.4	—
III-15/F/33	24	9	OU: ND	OD: 0.6	—
				OS: 0.1	—

However, some patients such as III-4 of family A (Figure 2B) and II-5 of family C (Figure 2C) showed quite atypical features different with typical LCD I. These patients showed a diffuse opacity both in the corneal epithelium and stroma, occupying the central cornea with superimposed amorphous deposits. The tiny intraepithelial microcysts or vesicles were similar to Meesmann's corneal dystrophy (Meesmann’s CD), which affects the corneal epithelium. Both patients had once been diagnosed with Meesmann’s CD when they went to the hospital for the first time. Even more interesting is that patients III-4 from family A also manifested thin branching refractive lines in the anterior corneal stroma (Figure 2B).

The proband of family B (Figure 2D), a 43-year-old man, was the most severe patient. He showed a diffuse opacity of throughout the cornea, however, no lattice lines could be found. Neovascular infiltration from the limbus of the cornea could be seen by slit lamp microscopy. He also had painful bilateral recurrent corneal erosions and visual defects since he was 13 years old, and such symptoms progressively aggravated. He first presented at our hospital when he was 30 years old with severe symptoms where the visual acuity of his right eye was hand movement (HM). Slit lamp examination of the right cornea did not reveal any lattice lines but a diffuse opacity all over the cornea following the recurrence of corneal erosions. He had been diagnosed with corneal ulcer and had received long-term antibiotic therapy elsewhere. However, his elder sister (III-2) manifested clinical features of typical LCD I.

### DNA analysis

**Figure 3:**
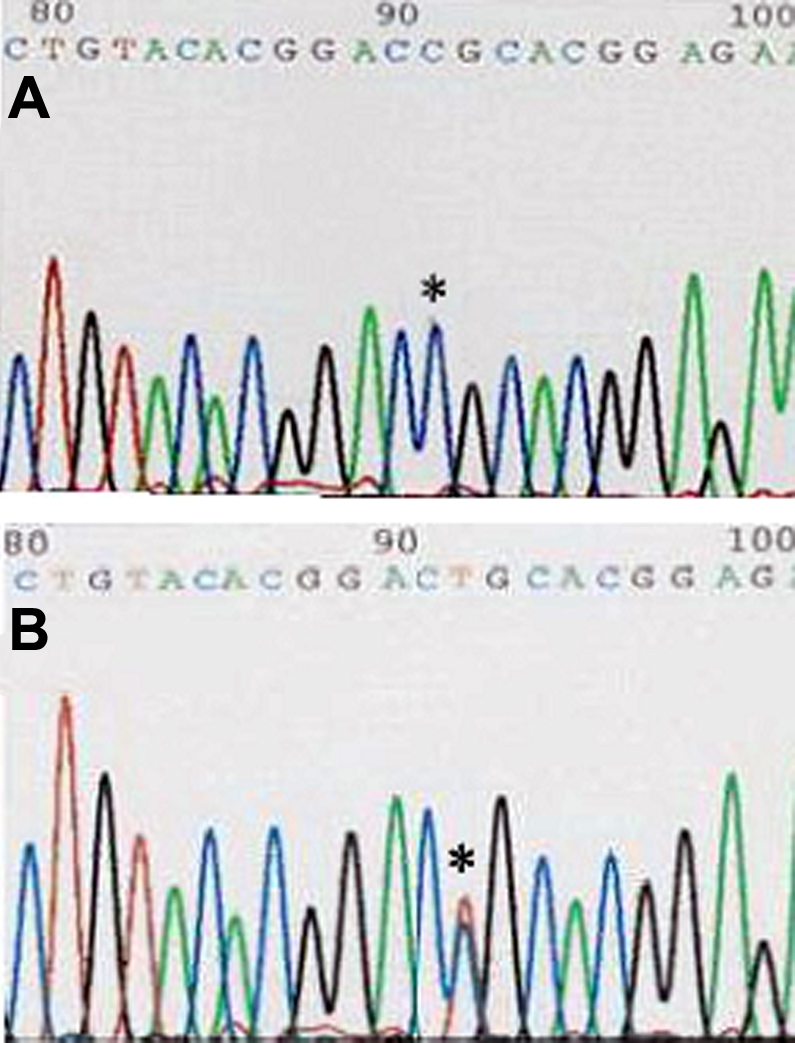
Results of direct sequence analysis of exon 4 of *TGFBI* in the region encompassing codon 124. The asterisk indicates that the first base of codon 124 in affected family members showed both red (T) and violet (C) peaks resulting in a single base-pair transition leading to an amino acid substitution, Arg124Cys (bottom panel). No equivalent mutation was detected in the control subjects (top panel).

Direct sequencing of *TGFBI* using the automated DNA sequencer revealed that 19 affected individuals and two young asymptomatic relatives showed the same mutant pattern whereas 15 unaffected showed the wild type pattern. The result was confirmed using both sense (S) and antisense (A) primers. The repetition of the sequencing confirmed the mutation. A single base-pair transition in exon 4 of *TGFBI,* leading to an amino acid substitution (C417T, CGC→TGC, Arg124Cys) was detected in all 19 affected individuals. Figure 3 showed examples of the heterozygous LCD I-associated mutation of affected patients, and no genetic defect was recognized in the unaffected individuals.

*KRT3* on chromosome 12q13 and *KRT12* on chromosome 17q12 has been found to be the main cause of Meesmann’s CD [12,13], the fact that all these patients were identified with a heterozygous R124C mutation in *TGFBI* corrected the former diagnosis of these patients with atypical features.

## Discussion

Lattice corneal dystrophy is generally divided into three subtypes. The classification has been made mainly by clinical and pathological findings. LCD I is characterized by prominent delicate linear opacities that tend to be mainly in the superficial corneal stroma accompanied with epithelial erosions.

During the past decade, several autosomal dominant hereditary corneal dystrophies had been related to mutations in *TGFBI/BIGH3*. Nearly 30 different mutations in *TGFBI* have been identified in families with variants of LCD [11,14-20]. Therefore, due to such genetic heterogeneity, it is necessary to identify which specific mutations are carried by the patient to assess a final diagnosis. However, in clinical practice, we may occasionally encounter patients with atypical clinical features of corneal dystrophy, which is difficult to diagnose by clinical features only.

In this present study, we report three Chinese pedigrees with variable clinical features where misdiagnosis had been made in some of the patients. Since phenotypic variations within these families are apparent, some patients’ clinical features of lattice corneal dystrophy are quite atypical. For this reason, classifications of the phenotype for these patients are very difficult. Genetic analysis of *TGFBI* showed that all affected members of the family have a heterozygous mutation of R124C, which suggests they belong to a classification of LCD I. They are classified into LCD I by the genetic findings, as this mutation occurs independently in several ethnic groups.

Meesmann’s CD is a rare autosomal dominant corneal dystrophy that preferentially affects young family members. Therefore, the penetrance of Meesmann’s CD should also be nearly 50%. However, from these Chinese families, the penetrance of atypical LCD I patients resembling Meesmann’s CD is only 5.6%, which does not support the diagnosis of Meesmann’s CD. Furthermore, the probability of two mutations occurring in both *TGFBI* and *KRT3* (or *KRT12*) in two unrelated Chinese families should be extremely rare. To our knowledge, there is no such report in other countries. Although we had not done DNA analysis for *KRT3* and *KRT12* in the atypical LCD I patients resembling Meesmann’s CD, we prefer the diagnosis of atypical LCD I rather than Meesmann’s CD.

This is the first report of phenotypic variability with the R124C mutation in Chinese LCD I pedigrees. Other reports about atypical LCD I with R124C mutation have been found in a Greek family, a Japanese family, and a Bangladeshi family, which indicates that atypical clinical features could also happen independently in different ethnic origins [21-23]. Therefore, to speak of the clinical features of LCD I, we should keep in mind that it may include two subtypes, typical and atypical LCD I, even within the same family. The mechanism of the phenotypic variability with the same mutation and even within the same family still remains unknown and needs further study.

Currently, genetic diagnosis plays an important role in establishing the classification of corneal dystrophy. Molecular genetic analysis could establish reliable clinical diagnostic criteria and could improve the accuracy of clinical diagnosis. The technique of DNA diagnosis could also make prenatal and postnatal DNA diagnosis possible in our clinical practice. Once the Arg124Cys mutation is detected in an individual from these families at birth, it can be expected that he or she will develop corneal lattice dystrophy, which may require keratoplasty in the future.
